# Cytotoxic Induction and Photoacoustic Imaging of Breast Cancer Cells Using Astaxanthin-Reduced Gold Nanoparticles

**DOI:** 10.3390/nano6040078

**Published:** 2016-04-20

**Authors:** Subramaniyan Bharathiraja, Panchanathan Manivasagan, Nhat Quang Bui, Yun-Ok Oh, In Gweon Lim, Suhyun Park, Junghwan Oh

**Affiliations:** 1 Department of Biomedical Engineering and Marine-Integrated Bionics Research Center, Pukyong National University, Busan 608-737, Korea; sbrbtc@gmail.com (S.B.); manimaribtech@gmail.com (P.M.); si565@hanmail.net (Y.-O.O.); 2 Department of Biomedical Engineering and Center for Marine-Integrated Biotechnology (BK21 Plus), Pukyong National University, Busan 608-737, Korea; nhatquang85@gmail.com; 3 Department of Mechanical Engineering, Myong-Ji University, San 38-2, Nam-dong, Cheoin-gu, Yongin-si, Gyeonggi-do 449-728, Korea; 4 Department of Biomedical Engineering, University of Texas at Austin, Austin, TX 78712, USA

**Keywords:** astaxanthin, breast cancer, gold nanoparticle, cytotoxicity, photoacoustic imaging

## Abstract

Astaxanthin, a kind of photosynthetic pigment, was employed for gold nanoparticle formation. Nanoparticles were characterized using Ulteraviolet-Visible (UV-Vis) spectroscopy, transmission electron microscopy, and X-ray diffraction, and the possible presence of astaxanthin functional groups were analyzed by Fourier transform infrared spectroscopy (FTIR). The cytotoxic effect of synthesized nanoparticles was evaluated against MDA-MB-231 (human breast cancer cells) using a tetrazolium-based assay, and synthesized nanoparticles exhibited dose-dependent toxicity. The morphology upon cell death was differentiated through fluorescent microscopy using different stains that predicted apoptosis. The synthesized nanoparticles were applied in ultrasound-coupled photoacoustic imaging to obtain good images of treated cells. Astaxanthin-reduced gold nanoparticle has the potential to act as a promising agent in the field of photo-based diagnosis and therapy.

## 1. Introduction

Nanotechnology is a promising region in the field of biomedical research with increasing applications. Gold nanoparticle (AuNPs) is a domain in nanomedicine with wide applications including cancer treatment, drug delivery, immune assays [1], biomedical imaging [2], diagnosis, radiotherapy, hyperthermia, and photodynamic therapy (PDT) [3]. Although there are many methods available to synthesize nanoparticles, the use of biological methods is becoming essential as the chemicals used in some of the other methods are potentially dangerous to the environment and biological systems [4]. Biological molecules can reduce metals to form nanoparticles that are suitable for treatment in biological systems. The distinct feature of nanoparticles is their unique size, which allows them to enter cells and even reach the genetic material inside the nucleus. Biologically reduced nanoparticles have biocompatible coated surfaces. In the present study, we attempted to synthesize AuNPs using astaxanthin as a reducing and coating agent.

Astaxanthin (3,3′-dihydroxy-β,β′-carotene-4,4′-dione) is a reddish-orange ketocarotenoid found in crustaceans [5], microalgae, plants, and some yeasts [6]. Its primary function is light absorption during photosynthesis. Astaxanthin has a potent antioxidant capacity and anticancer activity [7], and it can boost the immune response by stimulating immune cells [8]. The astaxanthin pigment has beneficial effects on the biological system, and the Food and Drug Administration has approved the use of this pigment as a food colorant in animal and fish feed preparation [9].

Photoacoustic tomography (PAT) is an emerging technology that allows for the imaging of cells or tissues using contrasting agents like pigments and nanoparticles [3]. AuNPs have been effectively used in different bioimaging systems for diagnostic purposes [10]. AuNPs can be used as a contrast agent for image-guided therapy. Fan [11] synthesized methylene blue-coated magnetic core AuNPs to image the lymph node metastasis in Caucasian male prostate (LNCaP) adenocarcinoma cancer cell line and used the same complex for photothermal and photodiagnostic therapies. In the present study, we synthesized AuNPs using astaxanthin and found an interesting UV-Vis absorption peak in the near infrared region (NIR). The NIR-absorbing nanoparticles are essential in photo-based diagnosis and therapy because the NIR laser can penetrate into tissue effectively and nanoparticles can convert NIR light into thermal energy, which is applied in photothermal therapy (PTT). In the present study we examined cytotoxic effect of synthesized nanoparticles and evaluated its imaging efficacy on breast cancer cells using 808 nm NIR illumination.

## 2. Results and Discussion

### 2.1. Synthesis and Characterization of Gold Nanoparticles

**Figure 1:**
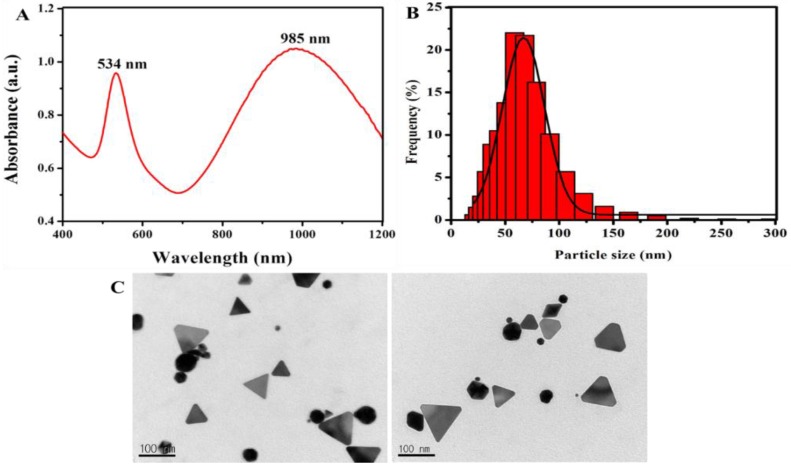
(**A**) Ulteraviolet-Visible spectrum of astaxanthin-reduced gold nanoparticles (Atx-AuNPs); (**B**) size distribution of Atx-AuNPs; (**C**) the topography of Atx-AuNPs represents major triangular and spherical shapes from the transmission electron microscopy (TEM) imaging. a.u.: absorbance units.

In the present investigation, AuNPs were formed by simply mixing astaxanthin with gold chloride solution without any external energy. The nanoparticles represented hereafter will be referred to as astaxanthin-reduced gold nanoparticles (Atx-AuNPs). Astaxanthin pigment has two terminal ring systems connected by double bonds of carbohydrate and displays strong antioxidant and anticancer properties [12]; in addition, it can act as a reducing biomolecule. Phytochemical-reduced AuNPs have great biocompatibility with promising anticancer effect and other biological applications such as biosensing, PTT, and PAT imaging [13]. A significant color change was observed within 40 min, and a surface plasmon resonance (SPR) band appeared at 534 nm and 985 nm (Figure 1A). Similarly, Klekotko reported the presence of one narrow SPR at 540 nm for spherical and one broad SPR around 900–1000 nm for anisotropic shapes like triangular and hexagonal AuNPs by green reduction using mint extract [14]. We are the first to report the synthesis of AuNPs using astaxanthin as a reducing agent. The appearance of optical response broad band around 985 nm in NIR was interesting, and nanoparticles of NIR absorption have potential applications in PTT, PDT, and PAT technology [15]. PAT, PDT, and PTT using NIR have the unique advantage of low systemic toxicity, remote controllability, and being able to image cancer cells in deeply situated tissue [16]. The transmission electron microscopy (TEM) image (Figure 1C) shows the presence of two major shapes of crystalline nanoparticles, spherical and triangular, which are corroborated by two SPR peaks. The sharp band at 534 nm and broad band around 985 nm correspond to spherical and anisotropic shapes respectively and size of the nanoparticles found to be in broad range from 30 to 250 nm. Based on dynamic light scattering (DLS) analysis, most of the particles’ size falls in the range of 60–120 nm (Figure 1B). Anisotropic shapes of nanoparticles includes majorly triangle found in Figure 1C may be responsible for the shift of UV-Vis spectrum absorption around 985 nm.

The SPR band is influenced by size, shape, morphology, and its interacting medium [17]. The present result shows the formation of a broad range of nanoparticles. It should be noted that the biological molecules used in nanoparticle synthesis process can also influence the SPR of bioreduced AuNPs [13,18,19]. Many studies manifested rapid synthesis of AuNPs using biological materials [18] having good biocompatibility with promising biological activities like antimicrobial and anticancer effects [19].

**Figure 2:**
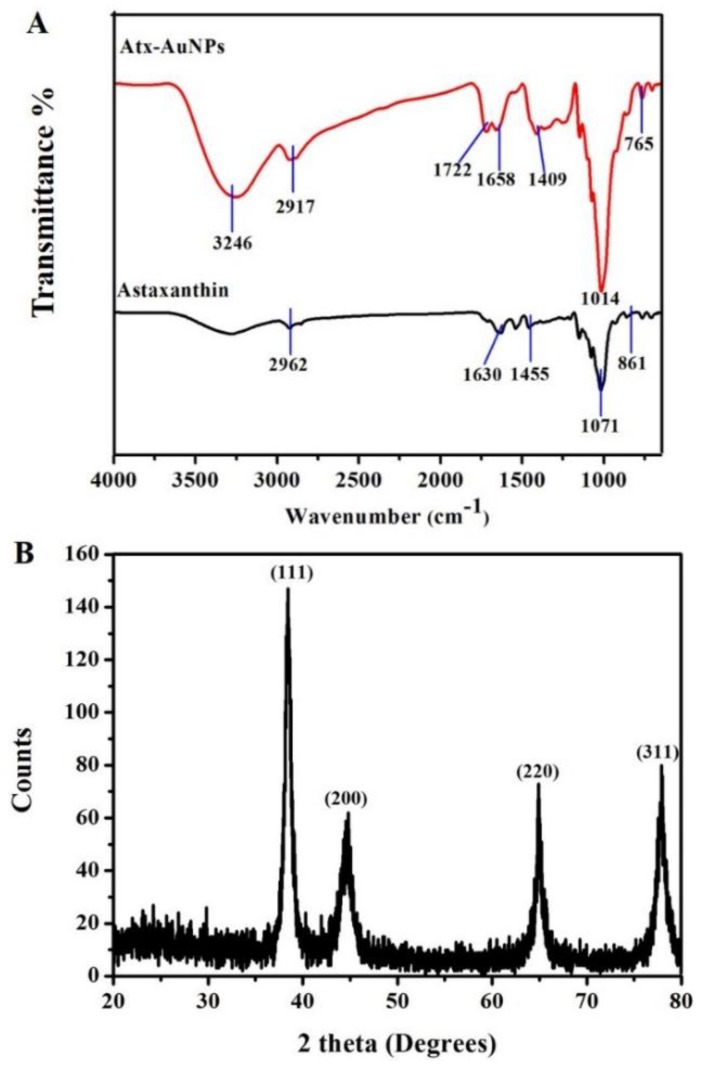
(**A**) Fourier transform infrared spectroscopy (FTIR) spectral analysis of astaxanthin and Atx-AuNPs; (**B**) The X-ray diffraction (XRD) pattern of Atx-AuNPs exhibits a strong Au signal.

FTIR analysis was performed to find the possible biomolecules of astaxanthin responsible for bioreduction or coating of Atx-AuNPs. Similar functional groups were found between interferograms of astaxanthin and Atx-AuNPs (Figure 2A). A strong band observed at 1071 cm^−1^ in astaxanthin and 1014 cm^−1^ in Atx-AuNPs is characteristic of –CH=CH_2_– bending, and the carbohydrate stretch acts as linker for the two aromatic rings in astaxanthin. A C–C stretch of aromatic rings was found at 1455 cm^−1^ and 1409 cm^−1^ in astaxanthin and Atx-AuNPs, respectively. The presence of aromatic functional groups in Atx-AuNPs was additionally confirmed by the peak at 765 cm^−1^, and the same group was found in astaxanthin at 861 cm^−1^. The peak at 1722 cm^−1^ in Atx-AuNPs indicated the involvement of carbonyl group in nanoparticle reduction. According to existing reports, carbonyl, hydroxyl, carbohydrate [20], and aromatic hydrocarbon [21] can reduce and cap AuNPs.

The X-ray diffraction (XRD) pattern further confirmed the presence of gold particles (Figure 2B). The intensities of crystalline AuNPs were recorded in XRD from 20° to 80°. The intense diffraction peaks at 2θ of 38.26°, 44.60°, 64.67°, and 77.54° corresponded to (111), (200), (220), and (311), respectively, and the pattern agreed well with the standard (JCPDS No. 04-0784) and earlier reports [22]. The peak assigned to (111) was stronger than the rest of the peaks.

### 2.2. Assessment of Cytotoxic Effect of Atx-AuNPs

**Figure 3:**
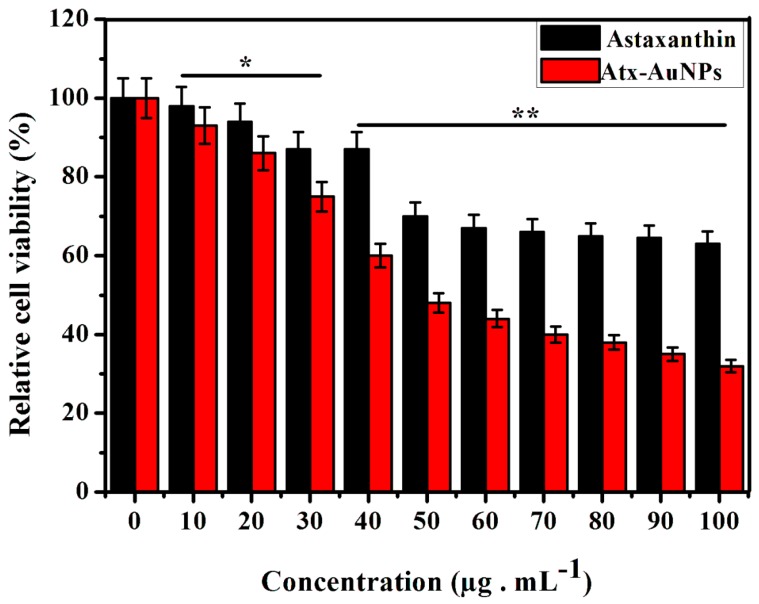
The 3-(4,5-Dimethylthiazol-2-yl)-2,5-diphenyltetrazolium bromide (MTT) assay experiment was performed in triplicate and the final values were represented as the mean ± standard deviation (SD). Legend: * = *p* < 0.05, ** = *p* < 0.01.

To assess the cytotoxic effect of synthesized Atx-AuNPs against Human breast cancer cell line (MDA-MB-231 cells), 3-(4,5-Dimethylthiazol-2-yl)-2,5-diphenyltetrazolium bromide (MTT) assay was performed with different concentrations ranging from 10 to 100 µg·mL^−1^ for 24 h. Atx-AuNPs showed effective antiproliferative effects with increasing concentration (Figure 3), and 50% inhibitory concentration was found as 50 µg·mL^−1^. They inhibited almost 60% of cell growth above a concentration of 80 µg·mL^−1^; however, they do not show much variation within the concentration range of 80–100 µg·mL^−1^. The cell viability increased when the doses decreased. The cytotoxic effect of astaxanthin is low when compared with Atx-AuNPs.

The use of AuNPs has increased significantly in medical applications. Previous studies reported that the cytotoxic effect of AuNPs will vary depend on concentration [23], synthesis methodology [24], and types of cell [25]. Generally AuNPs induce significant cytotoxicity in concentrations above 100 µg·mL^−1^. El-Kassas reported 80% of Michigan Cancer Foundation-7 cell viability at a 100 µg·mL^−1^ concentration of AuNPs [26]. In the present study we used 100 µg·mL^−1^ of Atx-AuNPs as the maximum concentration, which exerts 68% cell death.

### 2.3. Microscopic Analysis of Cell Death

**Figure 4:**
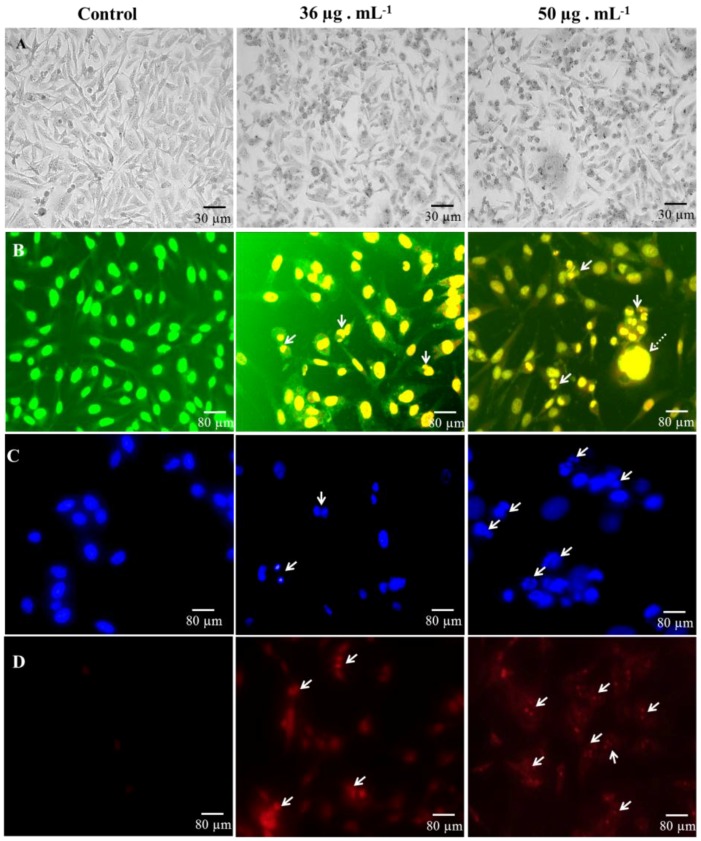
Bright field images of control and treated Human breast cancer cell line (MDA-MB-231 cells) (**A**). Fluorescence microscopy analysis of cell death using Acridine orange-Ethidium bromide (**B**); Hoechst (**C**); and Propidium iodide (**D**) stains. Arrows indicate the apoptotic cell morphology and the dashed arrow indicates necrotic cell death.

Bright field microscopic images show the difference between controls and treated cells. We can observe morphological changes like disturbed cell shape, growth inhibition, and cytoplasmic condensation in Atx-AuNPs-treated cells (Figure 4A), which are not seen in the control photograph; the cells remain live with a uniform structure. Atx-AuNPs-induced morphological alteration was observed using nucleic acid binding Acridine orange-Ethidium bromide (AO-EB) staining. Control cells appeared in a uniformly light green color. As control live cells exclude orange EB stain, greenish-yellow cells were not documented in control cells. Cells were very different in the treatment group compared to the control cells. Figure 4B showed cell shrinkage and nuclei condensation, which is a step of apoptosis. Clearly fragmented nuclei (arrow marked) were observed and cells were shrunken in the treatment groups. Some bulged necrotic cells were also observed (dashed arrows). AO-EB fluorescent staining allowed us to discriminate Atx-AuNPs-induced apoptotic cells from control cells.

We also used the following nuclei stains to differentiate cell death. Hoechst stain stains the nuclei of the cells regardless of their viability [27], allowing one to distinguish whether or not Atx-AuNPs caused the changes in nuclei morphology. Atx-AuNPs-induced nuclei condensation was photographed and represented by arrows in the treated cells (Figure 4C). The number of abbreviated and cleaved nuclei increased in the 50 µg·mL^−1^ dose treatment. The photograph of control cells shows an absence of punctate nuclei, and expressed cells remain normal.

Propidium iodide is a widely used nuclei staining that can enter into cells depending on the plasma membrane integrity, and therefore cannot stain living and early apoptotic cells [28,29]. Results from the PI staining indicate that the hallmark event of apoptosis, karyorrhexis (condensation of chromatin until it breaks into the cell) [30], was accelerated by Atx-AuNPs treatment (Figure 4D), which supports the results of the Hoechst staining. A round, condensed nucleus with red fluorescence was observed in nanoparticle-treated cells, indicated by arrows, and the nuclei of control cells remained unstained as PI cannot permeate the plasma membrane of viable cells.

### 2.4. Photoacoustic Image

**Figure 5:**
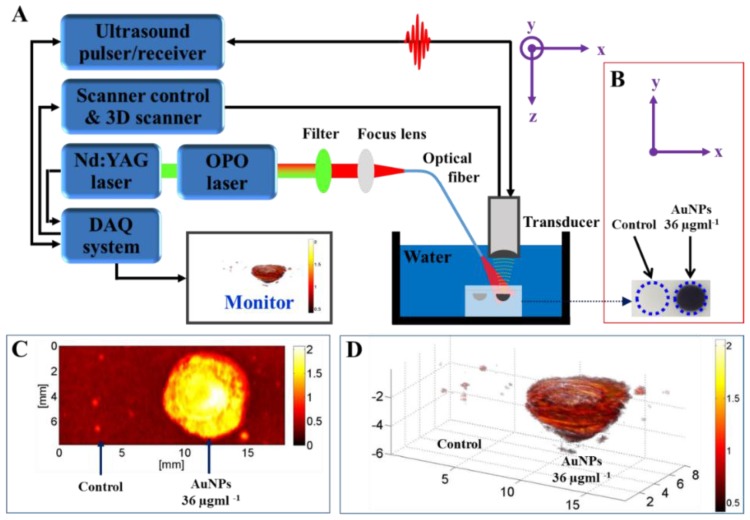
(**A**) Technical drawing of photoacoustic imaging system; (**B**) photograph of tissue-mimicking phantom; (**C**) maximum intensity projection (MIP) image and (**D**) 3 dimensional images of Atx-AuNPs-treated cells. Nd-YAG: neodymium-doped yttrium aluminium garnet; OPO: optical parametric oscillator; DAQ: data acquisition.

Tissue-mimicking phantom (control) and Atx-AuNP-treated cells are shown in Figure 5B. The maximum intensity projection (MIP) image along the Z-axis to the XY plane of the phantom is displayed over an 18 mm × 8 mm field of view (Figure 5C). We can observe the photoacoustic signal-generated image of Atx-AuNPs-treated cells; at the same time, control sample cells were not detected (Figure 5C). High-amplitude photoacoustic signals were detected from the inclusions of treated cells. The incident light was homogeneously distributed over the volume of cell inclusions, as observed in a 3 dimentional image (Figure 5D), which confirms the optical scattering property of Atx-AuNPs inside the cells. Figure 5D shows the 3D image of the phantom with an 18 mm × 8 mm × 6 mm field of view. Atx-AuNPs act as acoustic scatter, and a gelatin-based phantom provides the desirability of soft tissue. The scattered NIR results in hyperthermic expansion in the treated cells, which generates a broadband signal. Then, the signal was received by an ultrasound transducer to produce an image (Figure 5A). The gelatin-based phantom used here mimics the electromagnetic properties of biological tissue; this kind of *in vitro* phantom is rapidly emerging as an imaging technology used by many research groups [13]. This study shows that astaxanthin-synthesized nanoparticles can be used for photoacoustic imaging.

## 3. Materials and Methods

### 3.1. Reagents

Analytical grade chemicals were purchased from Sigma-Aldrich (St. Louis, MO, USA) unless otherwise mentioned and used without further purification. Gold (III) chloride trihydrate (HAuCl_4_·3H_2_O), astaxanthin, dimethyl sulfoxide (DMSO), and potassium bromide (KBr) were purchased from Sigma-Aldrich (St. Louis, MO, USA). All the cell culture reagents including Dulbecco’s modified eagle’s medium (DMEM), fetal bovine serum (FBS), penicillin, streptomycin, MTT, 1×trypsin, phosphate-buffered saline (PBS) were purchased from HyClone (South Logan, UT, USA) and staining reagents including acridine orange (AO), ethidium bromide (EB), Hoechst 33342, and propidium iodide (PI) were obtained from Sigma-Aldrich.

### 3.2. Formation of AuNPs

AuNPs synthesis was initiated by adding an equal volume of 0.001 M aqueous gold chloride solution to 0.002 M astaxanthin; the mixture was stirred at 250 rpm using a magnetic stirrer at room temperature for 24 h. Nanoparticle formation was observed through the changing of the solution to a pinkish color and was further monitored using UV-Vis spectroscopy (Beckman Coulter, Fullerton, CA, USA).

### 3.3. Characterization of Gold Nanoparticles

The reduced AuNPs mixture was centrifuged at 12,000*× g* for 30 min three times, and the resultant pellet was freeze-dried for FTIR and XRD analysis. An aliquot of the sample was pelletized with KBr for FTIR spectra generation by diffuse reflectance mode at a resolution of 4 cm^−1^ of wavelength about 4000–400 cm^−1^. The XRD spectra measured using XRD (X’Pert-MPD, Philips, Amsterdam, The Netherlands) with Cu-Kα radiation 1.5405 Å over an angular range of 5° to 80°, a step size of 0.02, a scan speed of 4°·m^−1^ at a 40 kV voltage, and a 30 mA current. To analyze the morphology of AuNPs, the aqueous sample was filmed on carbon coated copper grid, dried under an infrared lamp (JEM 1010 JEOL, Tokyo, Japan) (AC voltage −80.0 kV), and then mounted for TEM. An aliquot of liquid sample was analyzed by DLS to find the particle size distribution.

### 3.4. Cell Culture

MDA-MB-231 cells were cultured and maintained in DMEM containing 10% FBS and supplemented with 100 U·mL^−1^ penicillin and 100 µg·mL^−1^ streptomycin. The cultures were incubated at 37 °C in a humidified atmosphere with 5% CO_2_.

### 3.5. Antiproliferative Assay

The effect of Atx-AuNPs on breast cell viability was determined using a tetrazolium-based microplate assay. MDA-MB-231 cells were seeded in a flat-bottomed 96-well plate at a density of 1 × 10^4^ cells/well and kept in the CO_2_ incubator at 37 °C for 24 h for cell adherences. After incubation, the cells were treated with Atx-AuNPs dissolved in a plain medium at concentrations of 10, 20, 30, 40, 50, 60, 80, 90, and 100 µg·mL^−1^ in six replicates for 24 h. Thereafter, the medium from control and treated cells were removed and then 100 µL of MTT (0.5 mg·mL^−1^) dissolved in DMEM were added to every well. After 2–4 h of incubation at 37 °C in CO_2_ incubator, the MTT-containing medium was discarded and the formed purple formazan crystal inside the living cells was dissolved with 100 µL of DMSO. The developed purple color was measured in a microplate reader at 570 nm. Different concentrations of astaxanthin were used to analyze its cytotoxic ability. Initially astaxanthin was dissolved in 1 mL of 0.1% DMSO and treatment concentrations made with DMEM media. The relative percentage of cell viability was normalized with control cell viability and calculated using the following formula:

$$\%\text{ of cell viability}=\frac{\text{OD value of treated samples}}{\text{OD value of control samples}}\times 100$$

### 3.6. AO-EB Staining

Cells (1 × 10^5^) were seeded in a six-well plate and treated with different concentrations of Atx-AuNPs. Control cells were maintained without adding Atx-AuNPs. After 24 h incubation, cells were stained with mixture of fluorescent dyes (1:1 ratio) containing 100 µg·mL^−1^ AO and 100 µg·mL^−1^ EB and incubated at room temperature for 20 min before the stained cells was visualized (40× magnification) under a fluorescent microscope (Leica Microsystems GmbH, Wetzlar, Germany).

### 3.7. Hoechst 33342 Staining

Cells (1 × 10^5^) were cultured in a six-well plate and treated with different concentrations of Atx-AuNPs. Control cells were maintained without adding Atx-AuNPs. After a 24-h incubation, cells were washed with PBS and then fixed with 70% ice-cold ethanol for 10 min. The fixed cells were washed with PBS and stained with Hoechst 33342 (1 mg·mL^−1^) for 10 min. After washing off the excess dye with PBS repeatedly, images of cells were captured under a fluorescence microscope in the range of 450–490 nm.

### 3.8. Propidium Iodide Staining

MDA-MB-231 cells (1 × 10^5^) plated in a six-well plate were treated with Atx-AuNPs. The cells were washed with PBS and stained with PI stain (5 µg·mL^−1^) for 10 min at room temperature. The stained cells were washed several times with PBS and examined under a florescence microscope for nuclei of dead cells.

### 3.9. Tissue-Mimicking Phantom Experiment

A phantom that closely mimics human tissue was prepared for imaging of Atx-AuNPs-treated breast cancer cells using the PAT system. The tissue-mimicking phantom was fabricated by mixing 8% (*w*/*v*) polyvinyl alcohol (Sigma-Aldrich) with 0.4% (*w*/*v*) finely ground silica (Min-U-Sil, U.S. silica, Frederick, MD, USA) as an ultrasonic scattering agent in 100 mL of distilled water [31]. In total, 50 µL of cell inclusion consisting of Atx-AuNPs treatment and untreated control separately with gelatin solution was placed on the top of tissue-mimicking phantom. The rest of the gelatin solution filled up the phantom as the second layer after the solidification of the inclusions. Finally, the phantom was placed in the water tank of the photoacoustic imaging system to perform the imaging process.

### 3.10. Photoacoustic Tomography

The noninvasive photoacoustic imaging system has been previously developed and described by Bui [31]. The system integrated a pulsed neodymium-doped yttrium aluminium garnet (Nd-YAD) Q-switched laser (Surelite III, Continuum, San Jose, CA, USA) operating at 10 Hz repetition rate and 5 ns pulse duration, and an tunable optical parametric oscillator system (Surelite OPO Plus, Continuum, San Jose, CA, USA), which offers tuning from 650 to 1064. For photoacoustic imaging, an 800 nm wavelength was selected as the peak absorption of Atx-AuNPs. The free-space output laser beam was coupled to a 0.22 NA, 600 µm core diameter, multi-mode optical fiber (Thorlabs, Newton, NJ, USA) using a plano-convex lens of 50 mm in focal length (Thorlabs, Newton, NJ, USA). The input end of the optical fiber was fixed to a fiber coupler at the focal point of the lens. The output end of the fiber was combined to a 10 MHz single element, focused transducer (Olympus NDT, Waltham, MA, USA) and aligned such that the center of the irradiated light would be located at the focal point of the transducer. Both the output end of the fiber and the transducer were mounted on a 3D linear actuator for raster scanning. The output signals from the ultrasound (US) transducer were amplified using an ultrasound pulser/receiver (5900 PR, Olympus, Waltham, MA, USA). Then the signals were digitized and stored by a data acquisition (DAQ) system, which includes a 100-MS/s DAQ card (PXI-5122, National Instruments, Austin, TX, USA) and an embedded controller (NI PXI-1042Q, National Instruments). The DAQ system was synchronized with the trigger signals from the laser system to capture the Photoacoustic signals when the pulsed laser irradiated the sample. A custom-made LabView program (Version: 2010, National Instruments, Austin, TX, USA) was developed to control the DAQ system and the actuators. To form a PA image, the acquired PA signals were post processed to improve image quality by applying bandpass filtering (3–20 MHz) to reduce noise. The Hilbert transform was used to detect the signal envelope by taking the absolute values of analytic signals. Finally, the envelope signals were used to construct a 3D PA image using a home-made Matlab program (Version: 2013, MathWorks, Natick, MA, USA).

### 3.11. Statistical Analysis

The MTT assay experiment was performed in triplicate and the final values were represented as the mean ± standard deviation (SD). The statistical software, origin (one-way analysis of variance), was used to estimate the statistical parameters.

## 4. Conclusions

AuNPs have been synthesized using astaxanthin as a reducing agent without applying any external energy. Triangular and spherical crystalline Atx-AuNPs were observed. The astaxanthin-mediated nanoparticles showed potent cytotoxic effects on breast cancer cells and apoptotic morphology was detected in treated cells. The biogenic Atx-AuNPs act as a good contrast agent and gave an image of breast cancer cells in the NIR range, which can be used in therapeutic monitoring. A detailed study on the mode of astaxanthin reduction and its interaction with AuNPs is needed for understanding and further developing this particle as an image-guided therapeutic agent and will be explored in future studies.

## Abbreviations

The following abbreviations are used in this manuscript:

Atx-AuNPs	Astaxanthin-synthesized gold nanoparticles
AuNPs	Gold nanoparticles
MDA-MB-231	Human breast cancer cell line
NIR	Near infrared region
SPR	Surface plasmon resonance
PAT	Photoacoustic tomography
